# Inserting With Care: Classification of Complications Related to Chest Drain Insertion

**DOI:** 10.7759/cureus.104645

**Published:** 2026-03-04

**Authors:** Aimee Lee Jones, Kim Tien Chah, William Swee Keong Khoo, Tamara Fisher, Anna Grant, Matan Ben David, John Avramovic

**Affiliations:** 1 General Surgery, Townsville University Hospital, Townsville, AUS; 2 Urology, Townsville University Hospital, Townsville, AUS; 3 Trauma, Townsville University Hospital, Townsville, AUS; 4 Upper Gastrointestinal and Hepatobiliary Surgery, Townsville University Hospital, Townsville, AUS; 5 General Surgery, James Cook University, Townsville, AUS

**Keywords:** chest drain, chest wall trauma, clavien-dindo classification, emergency chest drain, general trauma surgery, haemothorax, intercostal chest tube drain, major trauma, pigtail catheter, pneumothorax ptx

## Abstract

Background

Intercostal catheters (ICCs) are standard management for traumatic pneumothorax (PTx), haemothorax (HTx), and haemo/pneumothorax (H/PTx). Pigtail catheters inserted using the Seldinger technique have emerged as a recognised alternative. Insertion steps are well described through the ‘triangle of safety’ using the sterile technique. Complication rates for chest drains have been reported as a variable rate, but complication reporting varies considerably, and some studies may overstate the clinical significance of minor events that do not require intervention. The Clavien-Dindo (CD) classification provides a standardised, intervention‑based method for grading complications and may offer a more clinically meaningful assessment than descriptive categories alone. This study examines chest drain complications at a regional trauma centre, comparing both conventional categories (positional, insertional, and infective) and a CD‑based definition.

Methods

A retrospective audit of all adult trauma-related ICC and pigtail drain insertions at Townsville University Hospital between February 2020 and February 2025 using data acquired from the Townsville Trauma Service Trauma Registry Information System (TRIS) was undertaken. Complications were recorded using (i) conventional definitions (insertional, positional, and infective) and (ii) CD-based complications (CDI, CDII, CDIIIa, CDIIIb, CDIVa, CDIVb, CDV). Comparative analysis was also performed to identify factors associated with adverse events.

Results

A total of 117 chest drains were inserted into 101 patients during the study period, with 71 of those being ICCs and 46 pigtail drains. The mean injury severity score (ISS) was 20.6; 90.5% percent of chest injuries were blunt and 9.5% were penetrating. Drain insertion was mostly performed for PTx and HPTx (43.6% and 41.9%, respectively), with a further 10.2% inserted for isolated HTx. One ICC was inserted for a chylothorax (CTx). The overall complication rate using the conventional definitions was 40.2% (2 insertional, 44 positional, and 1 infective). Most positional complications were due to post-drain removal (HTx (16) and PTx (20)); however, only 30% of these required drain reinsertions. The rate of complications using the CD classification was 16.2% (1 CDl, 14 CDIIIa, 3 CDIIIb, 1 CDIVa). There were no deaths related to chest drain insertion. In haemodynamically stable patients, ICCs demonstrated a higher proportion of both conventional complications (51% vs 37%) and CD‑classified complications (21.2% vs 5.3%); however, neither difference reached statistical significance. Clinician specialty and seniority had no effect on complication rates.

Conclusion

This study provides an important addition to the limited Australian literature on complications following chest drain insertion. Reported complication rates following chest drain insertion for chest trauma are higher using a conventional classification than with the CD classification. The additional recorded complications in the conventional classification do not result in clinically significant morbidity or interventions. Severity‑based classification for complications using the CD framework provides a more nuanced representation of patient harm and may improve the quality of future research in this area. This study supports the continued selective utilisation of pigtail catheters as a safe and less invasive alternative to ICCs.

## Introduction

Chest drains are the standard management for moderate to large volume traumatic haemothorax (HTx) and pneumothorax (PTx) and are a core procedural skill among general surgeons, emergency physicians, and critical care doctors. The standard approach through the ‘triangle of safety’ using a sterile technique is well established but has a varied complication rate [1-5]. This variation highlights the importance of standardised complication reporting.

Previous studies have attributed high complication rates to non-surgical specialties, inadequate training and clinician inexperience [5-7]. Clinician surveys investigating the impact of seniority on complications identified insufficient knowledge of the ‘triangle of safety’ as a potential source of error [8,9].

Historically, definitions of chest drain-related complications have varied considerably across the literature, limiting the comparability of outcomes between institutions and over time. Bailey's seminal retrospective review provided the first structured classification system, categorising complications into insertional (lung or organ laceration, neurovascular injury), positional (malposition, obstruction, extrathoracic placement, post‑removal PTx or HTx), and infective (superficial site infection (SSI) or empyema thoracis) [10]. This framework has since been widely adopted and remains foundational in contemporary reporting. However, studies including this framework often deviate to some extent from the original. For example, Inaba et al. diverged from Bailey’s original framework by including pneumonia as a complication, despite its potential association with other injuries such as rib fractures or ventilator‑associated pneumonia [11]. They also classified post‑removal PTx and HTx as complications only when re‑insertion was required, a criterion not explicitly defined in Bailey’s original study [10,11]. Kong et al., while using insertional and positional definitions similar to Bailey et al. [10], excluded infection as a complication, despite empyema thoracis being one of the most serious adverse outcomes following chest drain insertion [5]. More recently, efforts to standardise chest drain complication reporting have expanded. Aho et al. proposed a refined nomenclature incorporating five major categories: insertional, positional, removal-related, infective, and equipment malfunction [12]. Subsequent studies, including a systematic review by Hernandez et al., have reiterated the difficulty of evidence-based approaches to improve the quality of chest drain insertions due to the variety of complication classifications used. They supported the need for consistent terminology to improve benchmarking, quality assurance, and inter-institutional comparison [13]. 

The Clavien-Dindo (CD) classification system is a widely used, standardised system grading surgical complications based on both severity and required intervention. Its widespread use can be attributed to its simplicity, objectivity and strong correlation with outcomes such as length of stay and post-surgical quality of life [14,15]. Despite its widespread use among surgical specialties, there are currently no studies using this classification system with regard to chest drain insertions. 

Australian data on chest drain complications remain limited, and reported rates vary considerably. A recent retrospective review from New South Wales (NSW), which applied the conventional insertional, positional, and infective categories of complications, identified an overall complication rate of 25% [7]. The authors proposed that increasing reliance on conservative management for PTx/HTx, combined with the dispersal of trauma presentations across multiple centres, may be reducing procedural exposure and proficiency, thereby contributing to higher complication rates. In contrast, a review from the Alfred Hospital, a high‑volume trauma centre in Melbourne, reported a substantially lower complication rate of 7.6% [2]. Notably, the authors highlighted an unacceptably high rate of empyema thoracis, prompting the introduction of a surgical checklist that subsequently led to a marked reduction in this complication [6]. Queensland shares similar system characteristics to NSW, with several major trauma centres and a comparatively lower trauma volume compared to centres such as the Alfred Hospital. To date, there are no reviews of chest drain complication rates in Queensland, Australia. As such, understanding chest drain complication patterns within Queensland is essential for informing training, standardised practice and procedural governance. 

Pigtail catheters vs large-bore intercostal catheters

Intercostal catheters (ICCs) inserted for trauma are conventionally large-bore (20 - 40 Fr) and are typically performed using an open (blunt‑dissection) technique, in contrast to pigtail catheters, which are smaller and inserted using the Seldinger technique. The optimal drain type and size remain debated. Large-bore ICCs have historically been favoured for facilitating rapid evacuation of air and blood, but patient discomfort and emerging evidence regarding outcomes have prompted reconsideration [16]. Pigtail catheters (typically 14 Fr) have demonstrated comparable efficacy in selected traumatic PTx cases, with several studies reporting reduced pain and similar failure rates [17-19]. A recent meta-analysis by Beeton et al. found that pigtail catheters were associated with favourable outcomes without compromising safety, challenging the long‑standing preference for large-bore ICCs [17].

Peri-procedural antibiotics

Infective complications, ranging from SSI to empyema thoracis, are well recognised following chest drain insertion. Earlier guidelines, such as the 2012 EAST recommendations, were inconclusive regarding routine prophylactic antibiotic use [20]. Within it, the authors refer to a 2004 randomised controlled trial (RCT) by Maxwell et al., which reported a low overall incidence of empyema and found no evidence that prophylactic antibiotics reduced this risk. Instead, the authors identified factors such as prolonged in‑dwelling times, contamination at the time of insertion, and injury characteristics, particularly penetrating trauma, as more influential contributors to the development of empyema [21]. Multiple subsequent meta-analyses have demonstrated that antibiotic prophylaxis significantly reduces the risk of post‑insertion empyema in trauma patients [22-25]. MacDonald et al. published a systematic review demonstrating a reduction in empyema rates following chest drain insertion, but on subgroup analysis found that this was only found to be significant in penetrating trauma, but not in blunt trauma [24]. The nature of a non-sterile, penetrating object entering the thoracic cavity may confound the complication rate of chest drains. A systematic review by Ayoub et al. included 1263 patients and found that empyema rates were lower in the prophylactic antibiotic group compared to the placebo group (1% vs 7.2%, *p* = 0.0001) [23]. This growing body of evidence has shifted practice in many centres toward routine administration of a single prophylactic dose. 

Suction versus gravity drainage

Following insertion, chest drains are universally connected to an underwater seal system to allow passive evacuation of air and fluid. However, the role of active suction remains contentious. While negative suction has been shown to reduce postoperative PTx in thoracic surgery patients, its benefit in trauma is less clear [26]. A meta-analysis by Feenstra et al. found minimal impact on duration of tube placement, hospital stay, or persistent air leak [27]. Similarly, a 2025 RCT by Arora et al. reported only modest reductions in tube duration (3 (IQR 2-3.75) vs. 5 (3-8.75) days, *p* < 0.001) and length of stay (5 (4-8.75) vs. 10 (6-16.75) days, *p* = 0.004), suggesting that routine suction may not be universally beneficial in trauma populations [28].

Townsville University Hospital (TUH) is a regional tertiary centre in North Queensland, servicing 20 hospitals and a referral population of ~700,000. Due to variable chest drain complication rates in Australia and limited Queensland data, we conducted a retrospective review of insertions to assess complications using conventional classifications and CD-based complication rates.

## Materials and methods

This retrospective study examined complications associated with trauma‑related chest drain insertion at TUH, Queensland, Australia. The study included all patients aged 16 years or older who underwent chest drain insertion for traumatic indications at TUH between February 2, 2020, and February 12, 2025. Drains inserted pre‑hospital or at referring hospitals were excluded to ensure consistency in procedural technique, documentation quality, and complication attribution.

All eligible cases were identified through a prospectively maintained trauma registry, established at TUH in February 2020, which served as the primary source for case identification throughout the study period. 

Data collection was performed retrospectively using the electronic medical record (timer) and the picture archiving and communication system (PACS). Trauma registry data provided patient demographics, such as age and gender, time of injury, and time of drain insertion. Additional variables extracted from ieMR included physiological parameters at the time of insertion, injury characteristics (mechanism, size, and type of injury, as well as the injury severity score), the clinical indication for drain insertion, drain characteristics, operator specialty and level of training, documented complications, and patient outcomes. All data were collated and managed using Microsoft Excel.

Complications were classified as either conventional complications or CD complications.

Conventional complications were defined in accordance with previously published literature and were divided into (i) Insertional complications: organ laceration or perforation, intercostal or long thoracic nerve injury, and intercostal vessel injury, (ii) Positional complications: extrathoracic catheter placement, non‑function due to malposition, dislodgement, persistent PTx following catheter removal, and catheter repositioning or reinsertion and (iii) Infective complications: SSI infection or deep infection (e.g. empyema).

CD complications were defined using the Clavien-Dindo classification, with grades I, II, IIIa, IIIb, IVa, IVb, and V recorded (Table 1) [14]. All chest drains that were re-inserted or repositioned were recorded as CD IIIa.

**Table 1:** Clavien-Dindo classification Source: Adapted from the text in [14]

Clavien Dindo Grade	Definition
Clavien-Dindo I	Any deviation from the normal postoperative course without the need for pharmacological treatment or surgical, endoscopic and radiological interventions. Acceptable therapeutic regimens are: drugs as antiemetics, antipyretics, analgesics, diuretics and electrolytes and physiotherapy. This grade also includes wound infections opened at the bedside.
Clavien-Dindo II	Requiring pharmacological treatment with drugs other than such allowed for grade I complications. Blood transfusions and total parenteral nutrition are also included.
Clavien-Dindo III	Requiring surgical, endoscopic or radiological intervention
Clavien-Dindo IIIa	Intervention not under general anesthesia
Clavien-Dindo IIIb	Intervention under general anesthesia
Clavien-Dindo IV	Life-threatening complication (including CNS complications) requiring intermediate care/intensive care unit management
Clavien-Dindo IVa	Single organ dysfunction (including dialysis)
Clavien-Dindo IVb	Multi-organ dysfunction
Clavien-Dindo V	Death of patient

Haemodynamic instability was defined using physiological parameters at the time of insertion and was defined as heart rate over 100 or systolic blood pressure less than 100 at the time of chest drain insertion. 

The size of injury was recorded as small, medium, or large based on the radiology report of imaging performed prior to chest drain insertion. Documentation of clinical tension pneumothoraces with no radiological evidence prior to drain insertion was categorised as large.

‘On suction’ was defined as a chest drain recorded as being on suction at any point during the admission. If it was not documented either way, it was assumed off suction (underwater seal only).

Antibiotics were considered peri-procedural if they were administered intravenously within one hour of chest drain insertion.

Statistical analysis

Data were analysed using Microsoft Excel version 16.71 (Microsoft Corporation, Redmond, USA) and Stata® 18.0 (StataCorp LLC, College Station, Texas, USA). Differences in complication rates between registrars and consultants were assessed using Fisher’s exact test. All remaining comparisons were assessed using the Pearson Chi‑squared test for independence. A *p*-value of <0.05 was considered statistically significant. 

## Results

Over a five-year period, 117 chest drains were inserted into 101 patients. The mean age of patients was 50±19, with 83% being male. The mean ISS was 20.6; 90.5% of chest injuries were blunt, and 9.5% were penetrating. Patient age, ISS and gender had no impact on complication rate using either definition.

Indication for a drain

Chest drains were most commonly inserted for PTx, followed by H/PTx and HTx only (Table 2). 

**Table 2:** Indications for chest drain insertion

Injury type	n (% total)
Blunt	106 (90.6%)
Penetrating	9 (7.7%)
Blunt and penetrating	2 (1.7%)
Haemothorax	12 (10.3%)
Pneumothorax	51 (43.6%)
Haemopneumothorax	49 (41.9%)
Chylothorax	1 (0.85%)
Haemodynamic instability (prophylactic, no chest injury found)	4 (3.42%)

Volume

Six (5.1%) chest drains were inserted for small volume injuries, 40 (34%) for moderate, and 62 (53%) for large. Twenty-seven (23%) drains were inserted for clinical or radiological tension. Seven drains did not have a recorded volume. Two drains were inserted for haemodynamic instability and were not found to have a PTx or HTx. 

Haemodynamic stability and drain type

Forty-six chest drains were inserted in unstable trauma patients (39%). ICCs were more likely to be inserted for haemodynamically unstable patients compared to pigtail catheters (38/46 vs 8/46, *p* = 0.00009).

Type of drain

Overall, ICCs were more commonly placed compared to pigtail catheters (Table 3). 

**Table 3:** Chest drain characteristics ICC: Intercostal catheter

Drain characteristics	n (% total)
Pre-hospital needle decompression	6 (5.1%)
Pre-hospital finger thoracostomy	14 (12%)
ICC	71 (60.7%)
Pigtail catheter	46 (39.3%)
Bilateral drains	10 (8.5%)
On suction at any time	65 (55.6%)
Gravity drainage only	49 (42%)
Peri-procedural antibiotics	58 (49.6%)

Suction

Suction was applied to 55.6% of drains. There was a trend towards a longer overall in situ time when drains were placed on suction (mean 3.74 days vs 2.73 days; *p* = 0.053). After adjusting for volume of injury (small, moderate, large), there was no statistically significant difference found between in situ time when placed on suction compared to gravity drainage (adjusted effect = +0.98 days; *p* = 0.071). Placing drains on suction did not reduce the post removal HTx rate (*p* = 0.960) or post removal PTx rate (*p* = 0.482). Three chest drains had incomplete or missing documentation regarding suction or gravity drainage. Suction had no effect on the drain reinsertion rate (9.23% vs 16.3%, *p* = 0.253).

Antibiotics

As shown in Table 3, 49.6% of patients received peri-procedural antibiotics. One patient had a documented infective complication, and this was a superficial infection requiring antibiotics only. There were no recorded events of empyema thoracis. Infective complication rates (1/117) were too small to be considered for analysis (Table 4). 

**Table 4:** Conventional complications Conventional complications using Bailey's definition of insertional, positional and infective complications [10]. Because a single drain could exhibit more than one positional issue, sub‑category counts are not additive; totals reflect drains with any positional complication. PTx: Pneumothorax; HTx: Haemothorax

Complication type	n (total %)
Insertional complications	2 (1.7%)
Organ laceration or perforation	1
Intercostal artery or nerve damage	1
Positional complications	45 (38.5%)
Extrathoracic drain placement	1
Non-function due to suboptimal position	10
Dislodgement	6
Post-removal PTx	20
Post-removal HTx	16
Repositioned drain	13
Reinserted drain	13
Infective complications	1 (0.85%)
Superficial infection	1
Deep, empyema thoracis	0
Total:	48 (40.2%)

Complication rates

The complication rate was 40.2% when using the conventional definition for complications and 16.2% using the CD-based classification (Table 4, Table 5).

**Table 5:** Clavien-Dindo complications ICC: Intercostal catheter; VATS: Video‑assisted thoracoscopic surgery; PTx: Pneumothorax Clavien-Dindo classification [14]

Clavien-Dindo based complications	n (% total)
Clavien-Dindo I	1 (0.85%)
Superficial, surgical site infection	1
Clavien-Dindo II	0
Clavien-Dindo IIIa	14 (12.0%)
Positional errors resulting in repositioning or re-insertion	14
Clavien-Dindo IIIb	3 (2.6%)
Intercostal artery laceration requiring thoracotomy	1
Second ICC required intraoperatively due to suboptimal positioning	1
VATS secondary to suboptimal drainage	1
Clavien-Dindo IVa	1 (0.85%)
Iatrogenic tension PTx	1
Clavien-Dindo IVb	0
Clavien-Dindo V	0
Total:	19 (16.2%)

Thoracic surgery

Three patients underwent video-assisted thoracoscopic surgery for retained HTx, two of which were delayed presentations to the hospital (four and 11 days). These two chest drains were considered a ‘failure of intervention’ rather than a complication from chest drain insertion or positioning and were not counted as complications. The remaining patient had a chest drain inserted within 72 hours and was included as a CDIIIb. Nine patients underwent thoracotomy, none of which related to complications following chest drain insertion.

Prehospital treatment

Two endotracheal tubes were inserted for chest drainage and were later replaced by ICCs, and for the purpose of analysis, were counted as ICCs. Pre-hospital finger thoracostomies and in-hospital thoracostomies had no impact on conventional (35.7% vs 41.7%, *p* = 0.881) or CD (7.1% vs 17.4%, *p* = 0.737) assessed complication rates. 

Overall, ICCs were found to have a comparable complication rate to pigtail catheters using either definition (Table 6). There was no statistically significant difference in complication rates whether ICCs or pigtails were inserted for large volume injuries (*p* = 1.0). ICCs and pigtails had equivalent positional errors (39.4% vs 37%, *p* = 0.787). 

**Table 6:** Comparison of complication rates using conventional vs CD classifications for ICC vs pigtail drains in stable and unstable patients. Comparison of ICCs and pigtails according to haemodynamic status (overall vs stable vs unstable). Values are presented as n (%). Statistical comparisons were performed using a two‑tailed Pearson chi-squared test for independence. ICC: Intercostal catheter; CD: Clavien Dindo

Complications	Conventional classification ICC versus pigtail (n, % total)	Clavien-Dindo ICC versus pigtail (n, % total)
Overall	30/71 (42%) vs 18/46 (39%) *p* = 0.74	15/71 (21.1%) vs 4/46 (8.7%) *p* = 0.11
Stable patients	17/33 (51%) vs 14/38 (37%) *p* = 0.21	7/33 (21.2%) vs 2/38 (5.3%) *p* = 0.072
Unstable patients	13/38 (34%) vs 4/8 (50%) *p* = 0.4	8/38 (21.1%) vs 2/8 (25%) *p* = 1

Haemodynamic stability and complication rates

There was no difference in complication rates between chest drains and pigtail catheters in stable patients using the conventional classification. However, ICCs had a higher CD complication rate than pigtails in stable patients, though the difference was not statistically significant. Complication rates were similar in unstable patients using either classification (Table 6). 

A large number of complications using the conventional classification were attributed to the presence of post removal PTx or HTx (Table 4). Of these, most incidents did not require drain reinsertion and were therefore not considered complications using the CD classification (Table 7). Excluding these cases, the conventional complication rate fell to 23.9%.

**Table 7:** Positional complications requiring intervention *Some cases had both post removal pneumothorax and haemothorax Post chest drain removal pneumothorax and haemothorax *n* (%) and incidence of chest drain reinsertion *n* (%).

	n (%)	Required reinsertion n (%)
Post-removal pneumothorax	20	8 (40%)
Post-removal haemothorax	16	4 (25%)
Total	30	9 (30%)*

Proceduralist

Most drains were inserted by Emergency Department doctors (44.4%), followed by Cardiothoracic Surgery (40.2%). There was no statistically significant difference in complication rate between specialties. There was no statistically significant difference in the complication rate between consultants and registrars when measuring conventional complications (54.5% vs 38.6%, *p* = 0.150) or CD complications (18.2% vs 15.8%, *p* = 0.310).

## Discussion

This retrospective review demonstrates that while complications related to chest drain insertion are common when measured using conventional definitions, the majority are of little clinical significance. Using conventional definitions, the complication rate reached 40.2%. However, when complications were reclassified using the CD system, the rate of clinically meaningful complications fell to 16.2%. Conventional reporting also fails to distinguish minor issues, such as drain repositioning, from serious complications like empyema thoracis. This mismatch in reported complication rates, coupled with the absence of meaningful stratification, suggests that conventional classification systems may substantially overstate morbidity.

The elevated conventional complication rate in this study was largely attributable to positional issues, particularly post‑removal events. Although post‑removal PTx and HTx were frequently detected, only a small proportion required drain repositioning or reinsertion. Excluding cases that needed no intervention reduced the overall conventional complication rate to 23.9%. These findings indicate that radiological detection of minor HTx and PTx after drain removal may overstate complication rates without corresponding patient morbidity.

In comparison, Heng et al. reported a lower complication rate of 7.5% in a retrospective audit at the Alfred Hospital, Melbourne [2]. It is unclear whether routine chest radiographs were performed after removal, or whether post‑removal PTx was only documented when re‑insertion was required. Additionally, post‑removal HTx was not considered a complication in this study. In contrast, at John Hunter Hospital, Newcastle, Alrahbi et al. documented a higher ‘error’ rate of 38%. However, their definition encompassed additional factors such as omission of antibiotic prophylaxis, insertion through a previous ICC site, and incorrect drain size. The actual morbidity associated with these categories was not explored [4].

The application of the CD classification to chest drain complications provides a clinically intuitive framework that better correlates with patient impact. Unlike conventional definitions, CD captures only those events that require pharmacological, procedural, or critical care intervention. In this cohort, serious insertional injuries and empyema thoracis were appropriately classified as higher‑grade complications, while minor events such as radiological findings of small residual PTx and HTx were not captured. 

Complication rates were comparable between ICCs and pigtail catheters when assessed using either the conventional or CD classification systems. Among haemodynamically stable patients, ICCs demonstrated a higher proportion of both conventional complications (51% vs 37%) and CD‑classified complications (21.2% vs 5.3%); however, neither difference reached statistical significance. In unstable patients, complication rates remained similar between drain types using either classification method (Table 6). The findings of this study support the continued use of pigtail catheters as a less invasive alternative to ICCs. 

Proceduralist specialty and seniority were not associated with differences in complication rates. This suggests that chest drain insertion remains a safe and appropriate core skill among registrars and consultants across emergency medicine, surgery, cardiothoracics, and critical care. This contrasts with previous studies that favour seniority as well as thoracic and general surgeons [4,5,7].

The role of suction following chest drain insertion remains controversial. In this cohort, the use of suction did not reduce the incidence of post‑removal PTx or HTx and was associated with a trend toward longer drain dwell time. After adjustment for injury severity, suction conferred no statistically significant benefit. These findings align with emerging literature questioning the routine application of suction and suggest that selective rather than universal use may be appropriate [29,30].

Despite only half of patients receiving peri‑procedural antibiotics, infective complications were rare, with only one superficial SSI and no cases of empyema thoracis. This rate was lower than that reported in the locally comparable study by Heng et al. (2.4%) and similar to international data, including the 1% infection rate described in the systematic review by Hernandez et al. [2,13]. While this may reflect good aseptic technique and relatively short drain dwell times, it also contributes to the mixed evidence surrounding prophylactic antibiotics. Earlier guidelines, such as the 2012 EAST recommendations, were inconclusive, whereas more recent meta‑analyses have demonstrated benefit primarily in penetrating trauma. Our findings, which were derived from a predominantly blunt trauma population, suggest that infection risk may be inherently low in this group, raising the possibility that routine prophylaxis may not be necessary for all patients. This interpretation is consistent with the RCT by Maxwell et al*. *[21], which found no reduction in empyema with prophylaxis in a mixed trauma cohort. However, given the very small number of infective events in our study, definitive conclusions cannot be drawn.

An important contribution of this study is its alignment with longstanding concerns in the literature regarding the heterogeneity of complication definitions. Bailey’s original classification system, while foundational, has been variably interpreted across subsequent studies, leading to inconsistent reporting of insertional, positional, and infective complications [10]. Our findings reinforce the limitations of this descriptive framework, particularly its inability to differentiate clinically trivial radiological findings from complications requiring intervention. This echoes the concerns raised by Hernandez et al. [13], who highlighted the difficulty of synthesising evidence across studies due to inconsistent terminology and classification systems.

This study also contributes to the ongoing debate regarding optimal drain type. While large‑bore ICCs have historically been favoured for traumatic HTx and H/PTx, emerging evidence, including the meta‑analysis by Beeton et al., supports the safety and efficacy of pigtail catheters in selected trauma populations [17]. Our findings are consistent with this evolving evidence, showing comparable complication rates between ICCs and pigtails, even in large‑volume injuries.

This study provides an important addition to the limited Australian literature on chest drain insertion by offering contemporary, locally relevant data on complication rates and contributing factors. Further studies should build on these findings through prospective, multi-centre studies that apply the CD framework to chest drain complications to determine whether its use improves reporting consistency and clinical relevance. 

Study limitations

This study has several limitations. Its retrospective design risks incomplete documentation and reporting bias. Some drains were repositioned without a clear indication, inflating complication rates. Comparisons between ICCs and pigtail catheters, and suction versus gravity drainage, may be affected by selection bias. Small, medium, and large volume injuries were based on radiology reporting and had no specific size criteria. Suction time could not be measured, with only a binary ‘suction at any time’ approach, limiting reliability. Nevertheless, the use of a prospectively maintained trauma registry, a clearly defined complication framework, and the application of both conventional and severity‑based classifications strengthen the validity of the findings.

## Conclusions

While chest drain-related complications are frequently reported using conventional definitions, many of these do not result in clinically significant morbidity. Severity‑based classification using the CD system provides a more accurate representation of patient harm and may improve the quality of future research in this area. This study supports the selective utilisation of pigtail catheters as a safe and less invasive alternative to ICCs. The use of peri-procedural antibiotics and de-emphasis on routine suction remains controversial and further studies are required to provide clinical recommendations. As trauma systems continue to evolve, adopting standardised definitions may help refine procedural governance and guide more targeted training. Future prospective, multi‑centre studies are needed to validate these findings and further clarify optimal practice in chest drain management.
